# GNSS Receiver Fingerprinting Based on Time Skew of Embedded CSAC Clock

**DOI:** 10.3390/s24154897

**Published:** 2024-07-28

**Authors:** Sibo Gui, Li Dai, Meng Shi, Junchao Wang, Chuwen Tang, Haitao Wu, Jianye Zhao

**Affiliations:** 1 School of Electronics, Peking University, Beijing 100871, China; gsb530@stu.pku.edu.cn (S.G.); shim21@stu.pku.edu.cn (M.S.); wangjc@stu.pku.edu.cn (J.W.); tcw@bupt.edu.cn (C.T.); wuhaitao@stu.pku.edu.cn (H.W.); 2 ZhongkeQidi Optoelectronics Technology Company, Beijing 100083, China; daili@qdgdz.net

**Keywords:** GNSS receiver, chip-scale atomic clock, device fingerprint

## Abstract

GNSS spoofing has become a significant security vulnerability threatening remote sensing systems. Hardware fingerprint-based GNSS receiver identification is one of the solutions to address this security issue. However, existing research has not provided a solution for distinguishing GNSS receivers of the same specification. This paper first theoretically proves that the CSACs (Chip-Scale Atomic Clocks) used in GNSS receivers have unique hardware noise and then proposes a fingerprinting scheme based on this hardware noise. Experiments based on the neural network method demonstrate that this fingerprint achieved an identification accuracy of 94.60% for commercial GNSS receivers of the same specification and performed excellently in anomaly detection, confirming the robustness of the fingerprinting method. This method shows a new real-time GNSS security monitoring method based on CSACs and can be easily used with any commercial GNSS receivers.

## 1. Introduction

**Figure 1:**
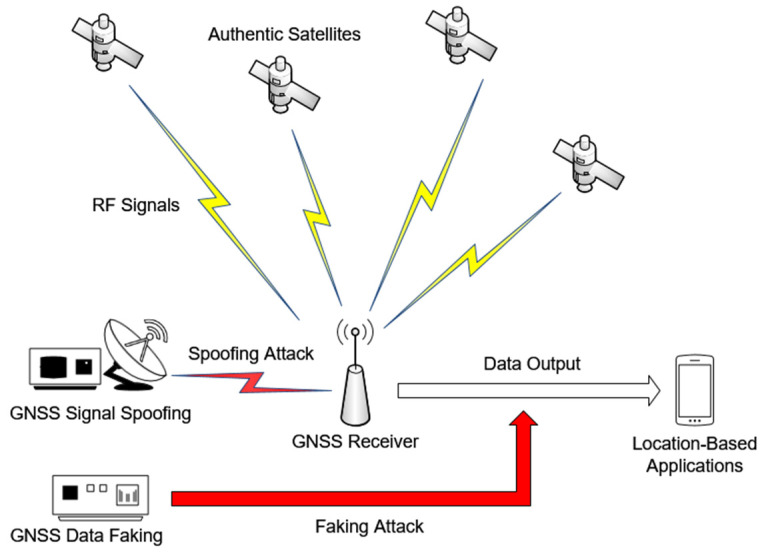
GNSS Receiver Attacks. The lightning symbol represents RF signals transmission, arrows represent data transmission. Red represents the methods of initiating an attack.

GNSS receivers provide positioning, navigation, and timing (PNT) information [1] that can serve complex systems, including geological monitoring [2], the Digital Tachograph (DT) for commercial vehicles [3], and the Automatic Identification System (AIS) for maritime applications [4]. These systems receive data transmitted by several pre-configured GNSS receivers and analyze it. Unfortunately, the transmission process is not secure. GNSS signal spoofing and GNSS data faking, collectively known as GNSS Receiver Attacks [5], can forge the information output by GNSS receivers, as illustrated in Figure 1.

When the satellite signals received by the GNSS receiver are replaced or forged, it results in signal spoofing, leading to erroneous PNT output. A typical case is the capture of an Unmanned Aerial Vehicle (UAV) [5]. By GNSS spoofing, Iran military successfully deceived a U.S. military UAV, causing it to land within Iran territory. Data faking occurs when the PNT information output by the receiver is altered during the process of network integration [6]. These security risks can be severe for remote sensing systems like AIS [7], making it such that ships are unable to seek true navigation information [5].

Electronic device fingerprints can be obtained by “gathering device information to generate device-specific signatures” [8]. Like human fingerprints, these device fingerprints can be used to determine the identity of a device when it interacts with other devices or accesses a network. For wireless devices, hardware fingerprinting is a well-established security technique [9]. Using unique RF fingerprints based on hardware intrinsic physical character, we can identify wireless devices and protect against various wireless network attacks, including spoofing and faking. Currently, device identification based on fingerprints is used for different purposes, such as intrusion detection [10], access control [11], clone detection, and secure localization [12]. Wireless platforms that use hardware fingerprints for device identification include HF RFID transponders [13], VHF transmitters [14], and IEEE 802.11 transceivers [15].

Due to the widespread presence of clocks in any radio devices, clock-based fingerprinting methods have been extensively researched [16,17]. Fabian Lanze et al. demonstrated that devices using different quartz crystal oscillators can be distinguished solely by clock skew—an unavoidable phenomenon that causes clocks to run at marginal but measurably different speeds—of these oscillators [18]. This passive hardware fingerprinting method measures the clock skew between the quartz oscillator’s signal and a more accurate lock, using it as the fingerprint of radio devices. This method is now widely applied in identifying computers [19], mobile devices [20], and IoT devices [21].

**Figure 2:**
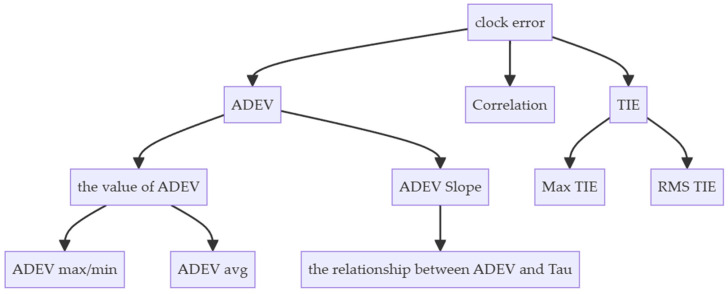
GNSS Receiver Fingerprint based on clock performance [22]. The application of statistical characteristics of the signal is a key aspect of this method. For example, Root Mean Square (RMS) and peak values, the relationship between Allan variance and Smoothing Time Interval (Tau), etc., are all taken into consideration.

GNSS receivers also embed reference clock sources, which can be directly compared with the satellite timing provided by the GNSS system to measure clock skew. As a result, researchers have begun exploring the feasibility of using clock skew fingerprints for GNSS receiver identification. Borio et al. pioneered the identification of various commercial and geodetic GNSS receivers by analyzing the statistical characteristics of clock deviations from the Time Compensated Crystal Oscillator (TCXO) embedded in GNSS receivers [22]. Local high-precision clock sources are supposed to help the fingerprint to be persistent [23,24]. Lin et al. studied high-precision GNSS receivers equipped with Micro Atomic Clocks and demonstrated that this method could also identify these receivers under both static and dynamic conditions. Their methods are as illustrated in Figure 2, using Allan Deviation (ADEV) [25], Time Interval Error (TIE), and self-correlation of the clock skew for fingerprinting.

However, current research relies on the performance of the reference clock embedded in GNSS receivers, particularly on the frequency stability characteristics represented by Allan variance. Frequency stability is not an intrinsic physical characteristic of the device but rather an artificially calculated external representation. Considering that previous work has focused on identifying the hardware devices produced by different companies under different technical conditions, using such characteristics is reasonable. However, this leaves a significant security loophole—how can we prevent spoofing using identical hardware models? This necessitates distinguishing GNSS receivers which embed clock sources from same specification and batch. Although, theoretically, there might be minor differences in the statistical characteristics of clock skew among clocks from the same batch and specification, further studies have confirmed that these skews are neither persistent nor stable and may change as the clocks age [25]. Therefore, the uniqueness and persistence of the fingerprint depending on the performance of clock are affected, highlighting the urgent need for a more stable and distinctive fingerprint.

This paper first derives that chip-scale atomic clocks (CSACs) have unique and unavoidable ultra-low-frequency noise in their clock skew due to deep-level defects in their semiconductor devices during production. Then, through experiments with three sets of commercial GNSS receiver from the same specification and batch, it demonstrates that clock skew information generated by CSACs, which are embedded in GNSS receivers, can serve as a fingerprint with uniqueness, persistence, and distinguishability. Specifically, we built a neural network for identifying GNSS receivers. This neural network is based on the Temporal Convolution Network (TCN) architecture and can identify features within the fingerprint information. Using the neural network, the accuracy of GNSS receiver identification reaches 94.88% and maintains high accuracy even when data quality declines and tasks become more complex, which proves the potential of this fingerprinting method in identifying GNSS receivers and detecting GNSS Spoofing.

## 2. Theory

### 2.1. CSAC Time Skew Analysis

**Figure 3:**
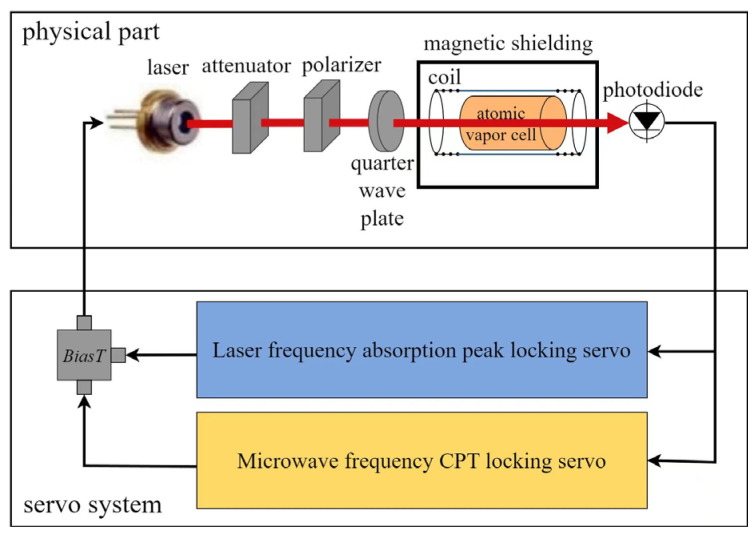
Chip-Scale Atomic Clock. Atoms in the vapor cell are trapped in a two-photon transition system due to CPT servo and can only absorb photons with specific energies. For more detailed information, please refer to [27].

Chip-Scale Atomic Clock (CSAC) is a high-precision clock source based on Coherent Population Trapping (CPT) [26]. It is miniaturized, consumes low power, and is highly stable. Its typical structure is shown in Figure 3. 

With similar volume and lower power requirements, CSAC’s frequency accuracy and aging rate are significantly improved compared to TCXO and other quartz oscillators, as illustrated in Table 1.

Therefore, replacing the quartz oscillators in GNSS receivers with CSACs as reference clock sources has been widely discussed and is believed to improve positioning accuracy while enhancing the robustness of GNSS receivers [28,29,30,31,32].

The clock skew of CSACs is extremely complex, influenced by temperature noise, vibration noise, VCSEL laser noise, and quantum noise. Currently, no comprehensive modeling and analysis of these noise sources exist. However, researchers have shown that laser noise is the primary source of noise in most Rb/Cs atomic frequency standards with lasers [33,34]. CPT atomic clocks use VCSEL lasers, which are microwave modulated in both amplitude and frequency by controlling their drive current, as described in Equation (1):

(1) $$S\left(t\right)=\left(1+\alpha \cos\omega_{m}t\right)A\cos\left\{\omega_{c}t+m\sin\omega_{m}t\right\},$$

where S(t) is the output light intensity of the VCSEL laser, $\omega_{m}$ is the angular frequency of the modulated microwave, $\omega_{c}$ is the laser carrier frequency, m is the modulation index, and α is the amplitude modulation index. Expanding Equation (1) using Bessel functions gives Equation (2):

(2) $$\begin{array}{c} S\left(t\right)=A\left(1+\alpha cos\omega_{m}t\right)\\ \cdot \{\begin{array}{c} J_{0}(\beta )\cos\omega_{c}t+\sum_{n=1}^{\infty }J_{2n}(\beta )[cos(\omega_{c}+2n\omega_{m})t+cos(\omega_{c}-2n\omega_{m})t]\\ -\sum_{n=1}^{\infty }J_{2n-1}(\beta )[cos(\omega_{c}+(2n-1)\omega_{m})t+cos(\omega_{c}-(2n-1)\omega_{m})t] \end{array}\} \end{array}$$

where $J_{n}(\beta )$ is the first kind of Bessel function. Equation (2) implies that the output light intensity of the VCSEL laser can be considered as the superposition of numerous monochromatic components.

On the other hand, due to the influence of the AC–Stark effect [35], the monochromatic laser causes a slight shift in energy level transitions, which can be shown as Equation (3):

(3) $$\Delta \omega_{i}=(1/4){\left|\omega_{iR}\right|}^{2}\frac{\Delta_{o}}{\Delta_{o}^{2}+\Gamma^{*2}/4}$$

where $\Delta \omega_{i}$ is the contribution of a monochromatic laser to the frequency shift. From Equation (2), the total frequency shift caused by all components of the VCSEL laser can be shown as Equation (4):

(4) $$\begin{array}{c} \frac{\Delta \omega_{LS}}{\omega_{0}}={\left(\frac{\omega_{R}}{\omega_{0}}\right)}^{2}\{\Theta (m)+\zeta (m)(\frac{\Delta_{o}}{\omega_{0}})+\xi (m){\left(\frac{\Delta_{o}}{\omega_{0}}\right)}^{2}\}\\ \Theta (m)=J_{0}^{2}(m)+(1/2)J_{1}^{2}(m)-2\sum_{n=2}^{\infty }J_{n}^{2}(m)(\frac{1}{n^{2}-1})\\ \zeta (m)\approx \alpha J_{1}(m)(J_{0}(m)+J_{2}(m))\frac{\omega_{0}^{2}}{\Gamma^{*2}/4}\\ \xi (m)=4J_{0}^{2}(m)+(1/2)J_{1}^{2}(m)-8\sum_{n=2}^{\infty }J_{n}^{2}(m)\frac{3n^{2}+1}{{\left(n^{2}-1\right)}^{3}} \end{array}$$

where $\Delta \omega_{LS}$ is the total frequency shift, $\omega_{0}$ is the eigen frequency of the atomic transitions, $\omega_{R}$ is the Rabi frequency, which is proportional to the square root of the laser intensity $P_{VCSEL}$, and $\Delta_{o}$ is the laser frequency detuning. Equation (4) shows that the frequency shift of CSAC can be considered as a quadratic function of the laser frequency detuning, with the coefficients of each term determined by the modulation index m as follows:

Traditionally, it is believed that if the external factors such as temperature can be controlled, the steady-state laser frequency detuning of the VCSEL $\Delta_{o}=0$ would cause the frequency to shift as follows:

(5) $$\frac{\Delta \omega_{LS}}{\omega_{0}}={\left(\frac{\omega_{R}}{\omega_{0}}\right)}^{2}\cdot \Theta (m)\;\propto \;P_{VCSEL}\cdot \Theta (m)$$

Equation (5) proves that time skew exists in the CSAC output signal, embodied as a frequency shift which is proportional to the VCSEL laser intensity. Considering that different CSACs often use different modulation index m, this can provide a basis for distinguishing between different brands and specifications of CSAC.

Furthermore, even if the external factors are controlled, intrinsic structural defects in the semiconductor part of the VCSEL laser, such as deep-level defects, will still cause instability by creating potential wells and continuously capturing and releasing carriers. Assuming the average recombination time of the jth deep-level defect with the carriers is $T_{j}$, we would approximate it as the current noise with a period of $T_{j}$. For all N deep-level defects, the following equation has been formulated:

(6) $$I_{VCSEL}\left(t\right)=I_{c}+\sum_{N}I_{j}\sin\frac{2\pi }{T_{j}}t$$

where $I_{VCSEL}\left(t\right)$ is the current inject in the VCSEL laser. The intensity of the VCSEL laser $P_{VCSEL}$, is given by $P_{VCSEL}=\mu_{d}(I_{VCSEL}-I_{th})$, where $\mu_{d}$ is the current gain.

(7) $$\omega_{LS}(t)\propto \;\Theta (m)\cdot (I_{c}-I_{th}+\sum_{N}I_{j}\sin\frac{2\pi }{T_{j}}t)$$

Equation (7) shows that the frequency shift of CSAC output signal is dependent on the deep-level defects in semiconductor materials. Due to the unique nature of deep-level defects in semiconductor materials, the frequency shift $\omega_{LS}(t)$ of different CSACs exhibits uniqueness. Considering that the time skew x(t) is the inverse short time Fourier Transfer with frequency shift $\omega_{LS}(t)$, different CSACs can be distinguished by measuring time skew.

On the other hand, extensive research has confirmed that semiconductor deep-level defects primarily arise during the semiconductor manufacturing process. For instance, semiconductor single-crystal materials typically generate numerous deep-level point defects (vacancies, anti-site defects, interstitials, and complexes) during growth, annealing, irradiation, ion implantation, and other processes. Compound semiconductors are prone to point defects due to inevitable deviations in chemical composition during growth. These defects do not change during the normal use of the semiconductor thus ensuring the persistence of these noise characteristics.

### 2.2. Fingerprinting Method

GNSS timing services typically update every second. Therefore, the collected time skew data can be regarded as a sampling of clock errors at a 1 Hz sampling rate, which can be considered as a time series. Therefore, fingerprinting based on clock skew can be viewed as a time series classification task. According to the Nyquist sampling theorem, signals above 1 Hz will cause frequency domain aliasing. To extract the unique signals described in the previous section from the time skew time series, we must first minimize high-frequency noise interference. Here, we used the Savitzky–Golay digital filter for smoothing. The principle is as follows:

Let x[i](i=−m,...0,...m) be the signal in a window length 2m+1. Now, construct an n−th order polynomial to fit this set of data as in (8):

(8) $$f\left(i\right)=\sum_{k=0}^{n}b_{nk}i^{k}$$

Then, calculate the fitting coefficients using the least squares method and, by continuously sliding the window, the smoothed values $x_{k,\mathrm{smooth}}$ at moment *k* can be calculated as in (9), in which $h_{i}/H$ be the coefficients calculated in the least squares method.

(9) $$x_{k,\mathrm{smooth}}=\bar{x_{k}}=\frac{1}{H}\sum_{i=-w}^{+w}x_{k+i}h_{i}$$

In recent years, with the continuous development of neural networks, network structures based on Convolutional Neural Networks (CNN) and Recurrent Neural Networks (RNN) have demonstrated their powerful feature extraction and classification capabilities in various time series tasks, including weather forecasting, electric power forecasting, and electroencephalogram (EEG) classification. These methods significantly outperform traditional algorithms like Support Vector Machine (SVM) and clustering. We used neural networks to classify the time skew series, thereby achieving GNSS receiver identification.

**Figure 4:**
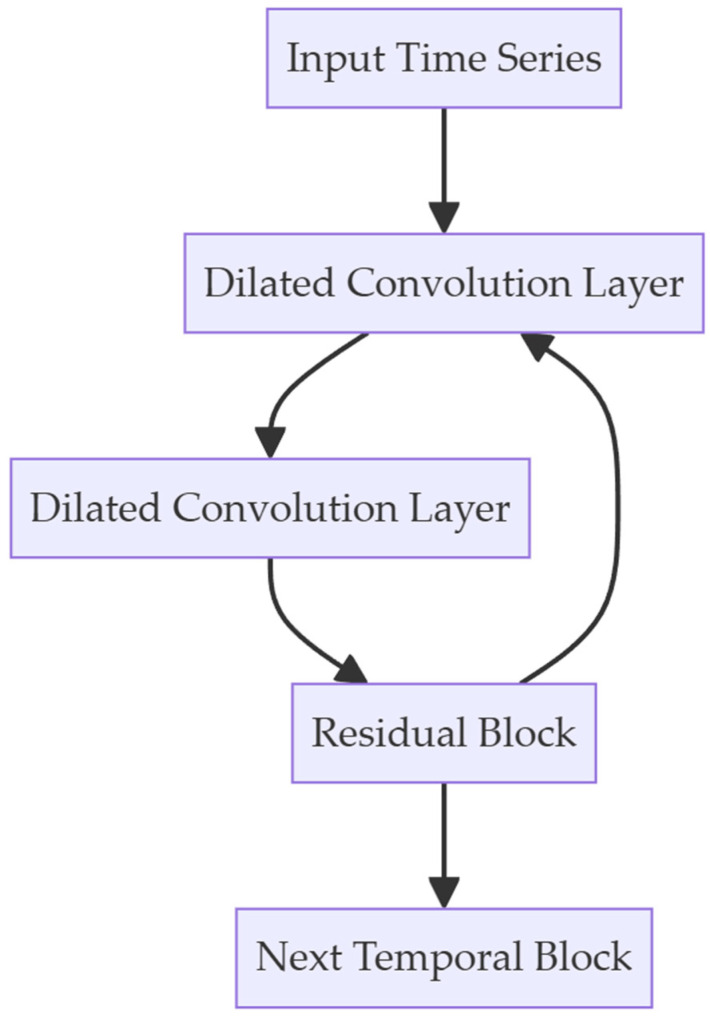
Temporal Block used in Temporal Convolutional Network. Dilated Convolution is used for extracting features in long term. For further information, please refer to [36,37].

Considering that GNSS receivers are often used in local area networks (LANs), where most devices are not specifically designed for computation and have very limited computational power and storage space, they may not support large-scale parallel computing. The network structures based on RNNs, due to their inherent computational logic requiring the storage of many intermediate states, are thus not suitable for adoption. Therefore, we used an improved structure based on CNN, namely the Temporal Convolutional Network (TCN), to build a fingerprint recognition model for devices within LANs. The main structure of the TCN network called the Temporal Block is shown in Figure 4.

## 3. Results

### 3.1. Experiment Platform and Data Collecting

**Figure 5:**
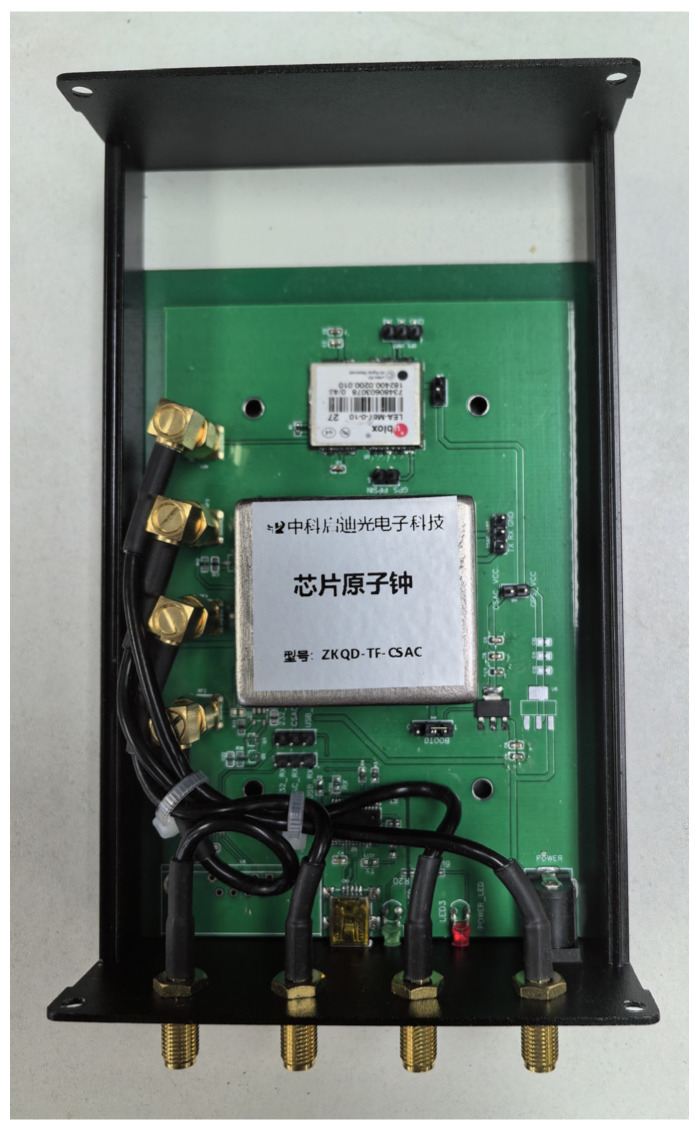
An example of the ZKQD-TF-CSAC embedded GNSS receiver, supplied by ZhongkeQidi Optoelectronic Technology (Beijing, China). Due to commercial secrets, we cannot show the newest version of this receiver. For more information, please see [38].

We used three commercial GNSS timing and positioning receivers provided by ZhongkeQidi Optoelectronic, equipped with high-precision positioning and timing modules (NEO-F9P and NEP-F10P, u-blox) and an embedded CSAC (ZKQD-TF-CSAC, ZhongkeQidi Optoelectronic) as a clock reference. These CSACs are from the same production line, with identical features. Figure 5 shows a CSAC from this batch.

**Figure 6:**
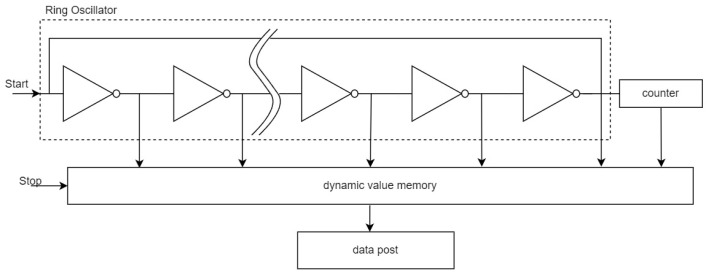
The structure of TDC module, including Ring Oscillator, Counter and Dynamic Value Memory. The Start and Stop signal serve as the start and end of Ring Oscillator.

To collect the skew of the clocks, we built a Time Digital Converting (TDC) measurement module, and the main principle is as shown in Figure 6. The CSAC and GNSS receiver output a 1pps signal as the TDC module’s Start and Stop signal. After triggering the measurement unit, the Start signal would oscillate in the ring oscillator and cause the counter to begin counting. Once the Stop signal is engaged, the position of the Start signal in the ring oscillator, the count value of the counter, and the delay of each logic gate can be used to calculate the time interval between the Start and Stop signals. 

Since voltage and temperature have a significant impact on the propagation delay time of gate circuits, errors caused by changes in temperature and voltage are usually compensated for through calibrated measurement methods. During the calibration process, the TDC module first measures one and two calibration clock cycles of the crystal oscillator and records the total counts as Cal1 and Cal2, respectively. Then, during the measurement of the time interval, the total counts HIT1 and HIT2 corresponding to the arrival of the two stop signals are recorded. The time interval between the two stop signals satisfies Equation (10):

(10) $$\begin{array}{ll} Time\;interval & =\frac{\left(HIT1-HIT2\right)}{\frac{\left(Cal2-Cal1\right)}{\left(2T_{ref}-T_{ref}\right)}}\\ & =\frac{\left(HIT1-HIT2\right)}{\left(Cal2-Cal1\right)}\times T_{ref} \end{array}$$

Because it only depends on the output clock signal, the TDC measurement module can be easily embedded in any commercial GNSS receiver which already uses a CSAC as a reference clock. For those which still use crystal oscillators, the TDC measurement module can be embedded with an external CSAC clock. For more detailed information, please see Appendix A.

From January to April 2024, we measured the clock skew of CSACs embedded in GNSS receivers during various weather conditions. The testing location was the laboratory of Peking University in Beijing, situated in an urban center with significant obstructions and multipath effects, simulating real-world application scenarios. To enhance data diversity, we also synthesized some simulated clock skew data by separately measuring the frequency shift of the GNSS receivers and CSACs before mixing them together. In total, we collected over 200 h of clock skew data for each CSAC embedded in the respective GNSS receiver. To ensure that the CSACs reached a stable operating state, we began data collection only after the GNSS receiver had been stably positioned for 5 h.

### 3.2. CSAC Time Skew Analysis

First, we verified whether the CSAC clock skew contained noise that could be used for fingerprinting as mentioned in Section 2.1. This noise should be unique, persistent, and of sufficiently low frequency to be completely sampled at a 1 Hz sampling rate. We began by converting the time-domain clock skew x(t) into frequency shift $\omega_{LS}(t)$ using the Hilbert transform, and then perform a Fourier transform to obtain $F_{LS}(f)$.

**Figure 7:**
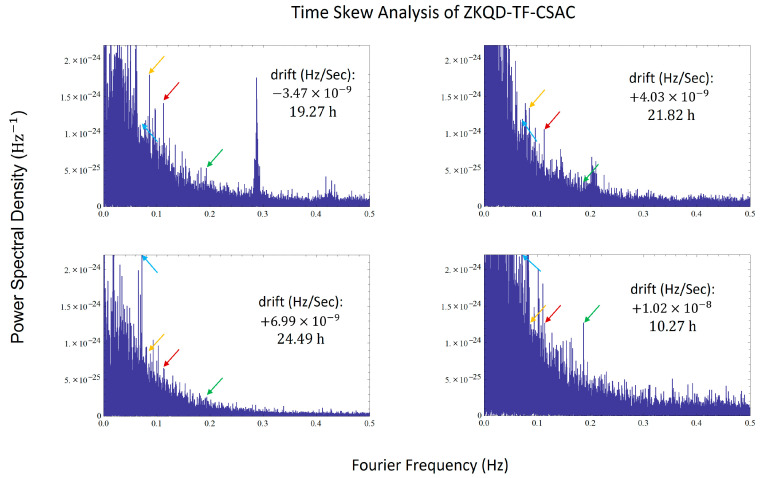
Time Skew Analysis of ZKQD-TF-CSAC, several spectral lines around 0.06 Hz, 0.08 Hz, 0.11 Hz, and 0.19 Hz reappeared in four different experiments with different conditions.

We first verified the persistence of the noise. Due to their smaller size and lower power, the CSAC’s vacuum performance was poorer, and their operating state was significantly affected by temperature and vibration, causing shifts in absorption peak position and microwave power. To simulate actual operating conditions, we conducted four tests in laboratory, indoor, outdoor, and field environments. The results are shown in Figure 7. Although the noise characteristics changed significantly, certain frequency combinations (indicated by arrows) consistently reappeared in the noise.

**Figure 8:**
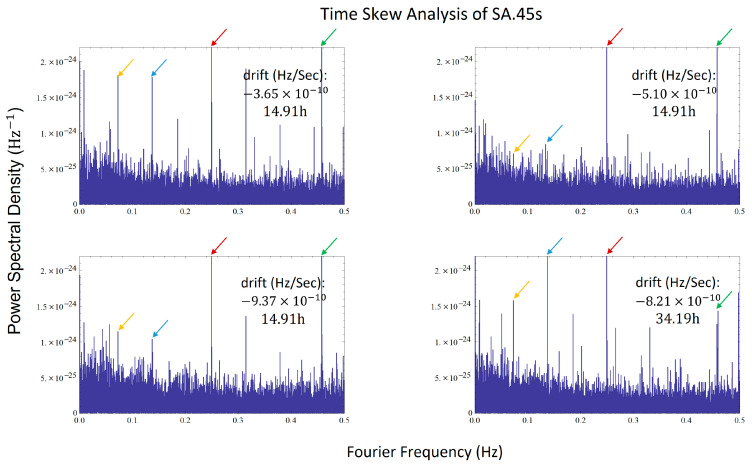
Time Skew Analysis of SA.45s, several spectral lines reappeared in four different experiments with different conditions, especially those around 0.25 Hz and 0.46 Hz, which S/N is more than 12 dB.

Noting that when the Fourier Frequency f→0, the Power Spectral Density increased rapidly. This can be attributed to the baseline drift of the CSAC’s frequency. For $\omega_{LS}(t)=\mu t$, its Fourier transform $F_{LS}(f)=-\frac{\mu }{j\omega }\cdot 2\pi \cdot \delta (f)$, where δ(f) is the Dirac delta function. This explains the rapid increase in the power spectral density as f→0. Therefore, we conducted tests using another CSAC with less baseline shift (Microsemi, sa.45s), and the results are shown in Figure 8.

Multiple noise spectral lines repeatedly appeared in the various experiments, confirming that there were indeed persistent ultra-low frequency lines in the noise of CSAC. These spectral lines exist in different operating states and when locking onto different absorption peaks, making them useful for distinguishing between different CSACs. Considering that these data were collected over a span of one year, this further demonstrates that the fingerprint features we used can maintain persistence over a long period.

Next, we verified the specificity of the noise. To demonstrate that the method was effective for all CSACs, we additionally measured two other types of CSACs with different technical approaches, in addition to the ZKQD-TF-CSAC. These were the XHTF1040 (Chengdu Spaceon Electronics) and the SA.45s (Microsemi). The technical differences are shown in Table 2.

**Figure 9:**
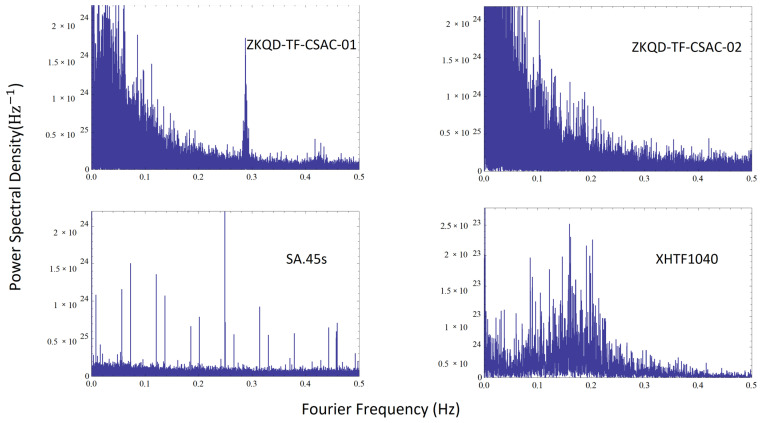
Time Skew Analysis of four different CSACs.

Two sets of ZKQD-TF-CSAC and one set each of SA.45s and XHTF1040 were used for comparison; we measured the clock skew of these four CSAC simultaneously to avoid environmental noise. The result is shown in Figure 9. The frequency domain characteristics of CSACs from different manufacturers exhibited distinct variations. These variations not only accounted for the differing performances observed in ADEV and TIE, which could explain other research, but also aligned with the theory in our research. This demonstrates that different brands of CSACs will produce completely different noise spectra and noise characteristics, making it feasible to distinguish them using time skew.

### 3.3. Fingerprint for Classification

**Figure 10:**
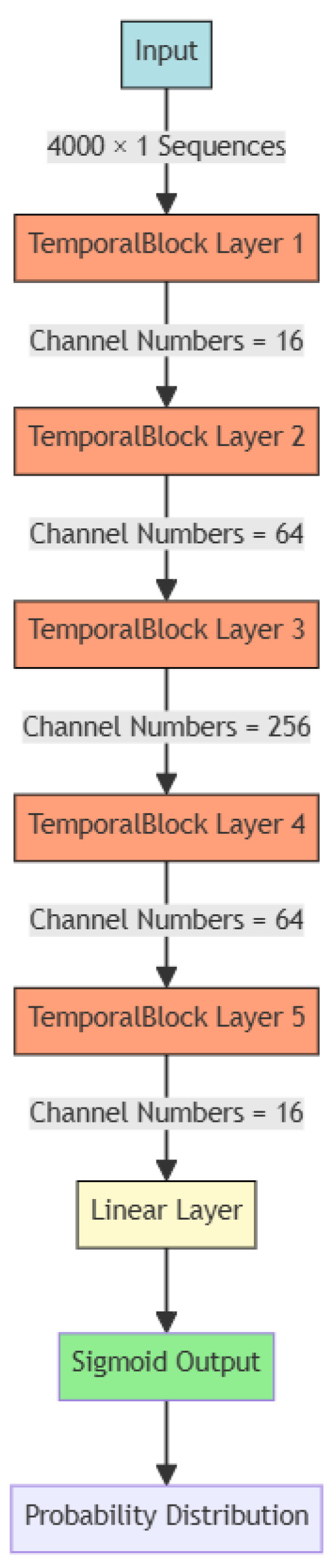
Fingerprinting Network. The number of input and output channels for each TemporalBlock Layer is given by the channel numbers specified between layers.

As described in Section 2.2, we first applied the Savitzky–Golay filter to denoise the clock skew. The denoised clock skew time series was used as fingerprint for each GNSS receiver. Next, we constructed a TCN network with the structure shown in Figure 10 and trained the network using clock skew data. The features were the clock skew time series, and the labels were the GNSS receiver IDs to which the clock skew series belonged. Considering that a typical GNSS spoofing attack requires approximately 4000 s, we segmented the clock skew into 4000 s sequences to observe whether the TCN network could identify the GNSS receiver to which these sequences belonged. A 5-fold cross-validation was used for dividing the training and test datasets. The classification accuracy for these three different commercial receivers was 94.60%, confirming that the TCN network could classify commercial receivers based on clock skew. This demonstrates the feasibility of using clock skew as a fingerprint for GNSS receiver identification.

**Figure 11:**
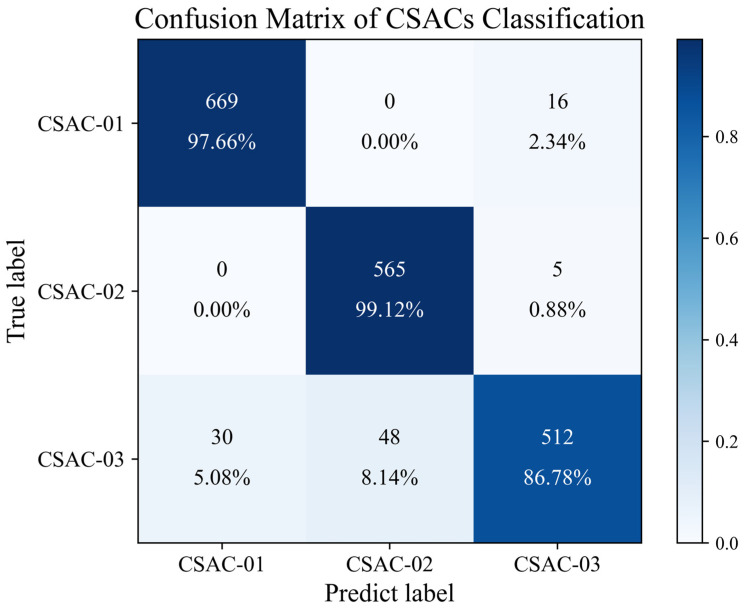
Confusion Matrix of CSACs Classification.

Figure 11 shows the confusion matrix for the classification task. It can be seen that the TCN Fingerprinting network performed well for all three receivers, demonstrating the potential of the fingerprinting method described in this paper for multi-target classification tasks.

## 4. Discussion

### 4.1. Impact of Time Skew Series Length

**Figure 12:**
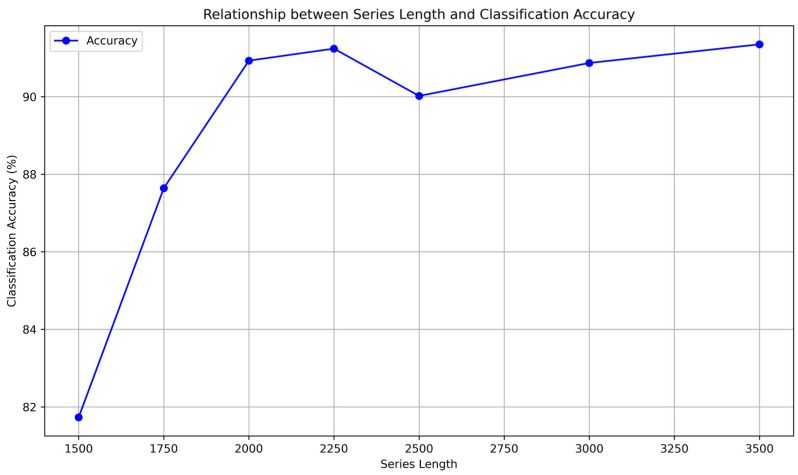
Relationship between Series Length and Classification Accuracy.

The 4000 s series length used in this study may lead to classification delays, posing significant risks in high real-time scenarios. Existing research indicates that the classification time for the operational status of atomic frequency standards can be reduced to 20 min [22,38], which would greatly enhance system security. This section will first discuss the feasibility of shortening the time needed for classification. We cut the time skew sequences to lengths of 1500 s–3500 s and retrained the TCN Fingerprinting network for classification. The accuracy rates are shown in Figure 12.

Figure 12 indicates that using 2000 s time series for classification can achieve an accuracy of over 90%, suggesting that our classification time can be reduced to 2000 s, which is suitable for most scenarios. On the other hand, when the time series length is further shortened, the classification accuracy rapidly drops to around 80%, which is inferior to traditional methods using Allan variance and TIE (approximately 1200 s) [39]. This indicates that further improvements in network structure and performance are necessary for future work.

### 4.2. Impact of Unstable GNSS Timing Services

Although theoretically the accuracy of GNSS time transfer (<10 ns) is superior to that of CSAC, making the clock skew between them primarily reflect the characteristics of CSAC, GNSS timing services can be affected by factors such as the number of satellites, weather, electromagnetic environment, and environmental conditions. These factors can sometimes cause interruptions or significant drops in timing accuracy (>500 ns), leading to timing anomalies that can contaminate the clock skew fingerprint. This issue is particularly evident in the field geodetic observation sites and in dynamic scenarios. In this section, we simulated this situation.

First, we collected timing errors from field GNSS observation sites provided by IGS and recorded the values that did not meet the timing accuracy standards, including cases with no timing information and those with low accuracy. Next, we randomly replaced the output of a stably operating GNSS receiver with these non-compliant timing values and compared it with the CSAC clock to simulate the clock skew between the GNSS receiver and CSAC under timing anomaly conditions.

**Figure 13:**
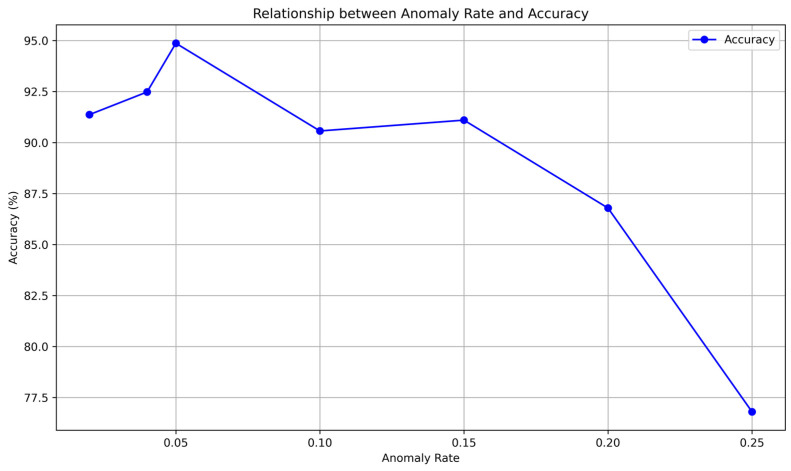
Relationship between Anomaly Rate and Classification Accuracy.

As shown in Figure 13, the model’s classification performance declines as the probability of data anomalies increases. When the anomaly rate is less than 5%, the model’s classification accuracy remains almost unchanged. However, when the anomaly rate rises to 25%, the model’s classification accuracy drops below 80%, rendering it ineffective. According to BDS and GPS documentation, the anomaly rate of their timing services is below 5%. Additionally, the receiver can further reduce the anomaly rate by receiving timing signals from multiple GNSS systems. Therefore, we conclude that timing service anomalies do not affect the fingerprint characteristics and the corresponding TCN network’s ability to identify GNSS receivers.

### 4.3. Feasibility for GNSS Spoofing Detection

Finally, we verified the feasibility of using our model for real-time GNSS Spoofing Attack detection. We assumed position at an outdoor monitoring site, where Geodetic GNSS Receiver 1 was normally transmitting PNT information to a remote endpoint, while a malicious actor performed data faking at T=10,000 s, altering the signal to be transmitted from Commercial GNSS Receiver 2 to the control terminal. Since the classification system operates continuously, it first received the concatenated fingerprint information from Receiver 1 and Receiver 2. We observed whether the TCN network could quickly identify the anomaly in such a sequence.

**Figure 14:**
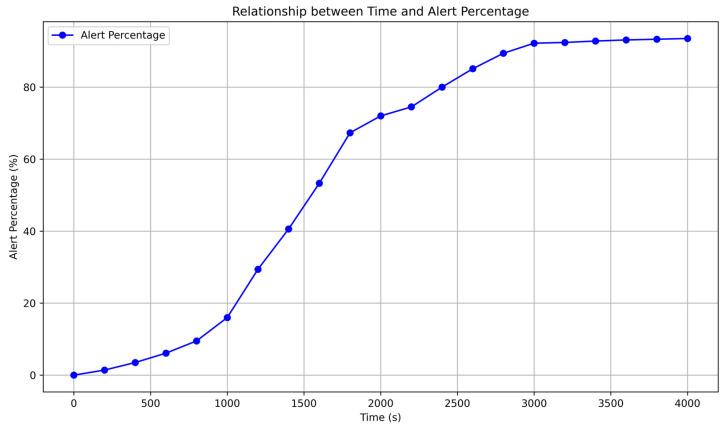
Relationship between Time and Alert Percentage.

To this end, we additionally collected time skew data from 20 CSACs and artificially generated time skew series. These data were uniformly labeled as “anomalous receivers” and used in conjunction with the previously mentioned data to train the TCN network. After training the network, we used the clock offset information from Receiver 1 as the input stream. This input stream automatically passed the clock offset information for the first 4000 s to the TCN. At this point, it was observed that the TCN could correctly classify the information. After 10,000 s, the input stream to the TCN was switched to Receiver 2, which the TCN was not previously trained on. If the TCN consecutively output abnormal classification results within 10 s, it was considered a sign that the TCN had detected spoofing. We recorded how long it took for the TCN to alert to an anomaly after time T, and the results are shown in Figure 14.

It can be seen that the TCN fingerprinting network could respond to spoofing within 2400 s in over 80% of cases. When the spoofing clock source was not the same atomic clock, the detection time could be significantly reduced. Using a 90% accuracy threshold, it took only 710 s to respond to spoofing attack based with SA.45s and just 265 s to respond to OCXO. This is sufficient to counter most GNSS spoofing attacks, indicating that the fingerprint and classification method proposed in this paper can also be used for anomaly detection, especially for GNSS systems where timing attacks are the primary security concern, such as large-scale power systems, BBUs in mobile communication networks, and large-scale geological disaster detection systems. This method can also provide security protection and supplementary safeguards for low-dynamic GNSS scenarios, such as maritime AIS systems and VT systems. This approach offers a stable and low-cost security solution for GNSS receivers.

## 5. Conclusions

Through experimental validation, this paper has proven that the fingerprinting method proposed in Section 2 is feasible and can serve as a key feature for classifying GNSS receivers under different conditions. Through demonstrative experiments, we validated the potential application of this feature in GNSS spoofing detection. This fingerprinting method fills the gap in previous research regarding the classification of clock sources of the same type and parameters, eliminating the risk of hardware cloning attacks on the fingerprint recognition of GNSS receivers, significantly enhancing the security of hardware fingerprints based on time skew in GNSS receivers.

Furthermore, this paper, through theoretical derivation and experimental verification, suggests that CSAC clock sources may be inherently tamper-proof, with time skew serving as an anti-counterfeiting identifier for CSACs. This further enhances the advantages of using CSACs over traditional quartz oscillators in various electronic devices. CSACs can play a greater role in remote sensing systems requiring security, such as AIS systems and geological monitoring systems. In particular, it can be combined with other security schemes, such as hardware fingerprints based on clock characteristics, intermediate frequency hardware fingerprints, and channel encryption, to form a more secure GNSS spoofing detection solution.

## Figures and Tables

**Table 1:** Typical parameters of TCXO and CSAC.

	TCXO (Freqtrol FCOX101)	CSAC (Microsemi sa.45s)
Size	21.0 mm × 13.6 mm × 8.5 mm	40.6 mm × 35.5 mm × 11.4 mm
Frequency Accuracy	3.0 × 10^−7^	5.0 × 10^−11^
Frequency Stability (Allan Variation, 1 s)	5.0 × 10^−11^	1.5 × 10^−10^
Aging Rate (per year)	5.0 × 10^−8^	1 × 10^−9^

**Table 2:** Technical Differences of CSACs.

	Atom Element	Chamber Technique
ZKQD-TF-CSAC	Rb	Cell
XHTF1040	Rb	MEMS
SA.45s	Cs	MEMS

## Data Availability

All CSACs and GNSS receivers’ data can be required by connecting the corresponding author for scientific use. Commercial use will need to obtain content from ZhongkeQidi Optoelectronic Technology Company.

## Appendix A

We achieved TDC (Time-to-Digital Converter) measurements with a precision of 22.5 ps by using a microcontroller to control a commercial TDC chip (TDC_GP21, ACAM). However, the range of commercial TDC chips is limited, typically around 1us, which is insufficient for long-term clock offset measurements. To address this, we used a microcontroller (MSP430) to monitor the TDC measurements in real time. When the clock offset exceeded the measurement range, the microcontroller intervened promptly to record the error between the two signals and realigned the falling edges of the two clocks, resetting the clock offset. This significantly extended the measurement range of the TDC module. For more information, please refer to [40]. The implementation accuracy of the TDC is a crucial indicator for the TDC module. To determine the implementation accuracy, it is first necessary to ascertain the error level of the clock offset and ensure that the measurement accuracy surpasses this level. For the chip clock used in this paper, the relative frequency deviation is approximately in the order of 10^−10^, making a measurement accuracy of 22.5 ps sufficient. However, for more precise atomic clocks, such as the Microsemi SA.5X series, an even higher accuracy TDC implementation may be required, such as using optical delay lines.
